# Supplementary material for: A Global Screen for Assembly State Changes of the Mitotic Proteome by SEC-SWATH-MS
Source: Cell Syst. 2020 Feb 26;10(2):133–155.e6. doi: 10.1016/j.cels.2020.01.001 (PMC7042714; doi:10.1016/j.cels.2020.01.001)

Q969M3 | YIPF5_HUMAN | YIPF5 FINGER5 YIP1A PP12723 SB140 UNQ3123/PRO10275
Monomer MW [kDa]: 27.989 Monomer expected elution fraction: 51

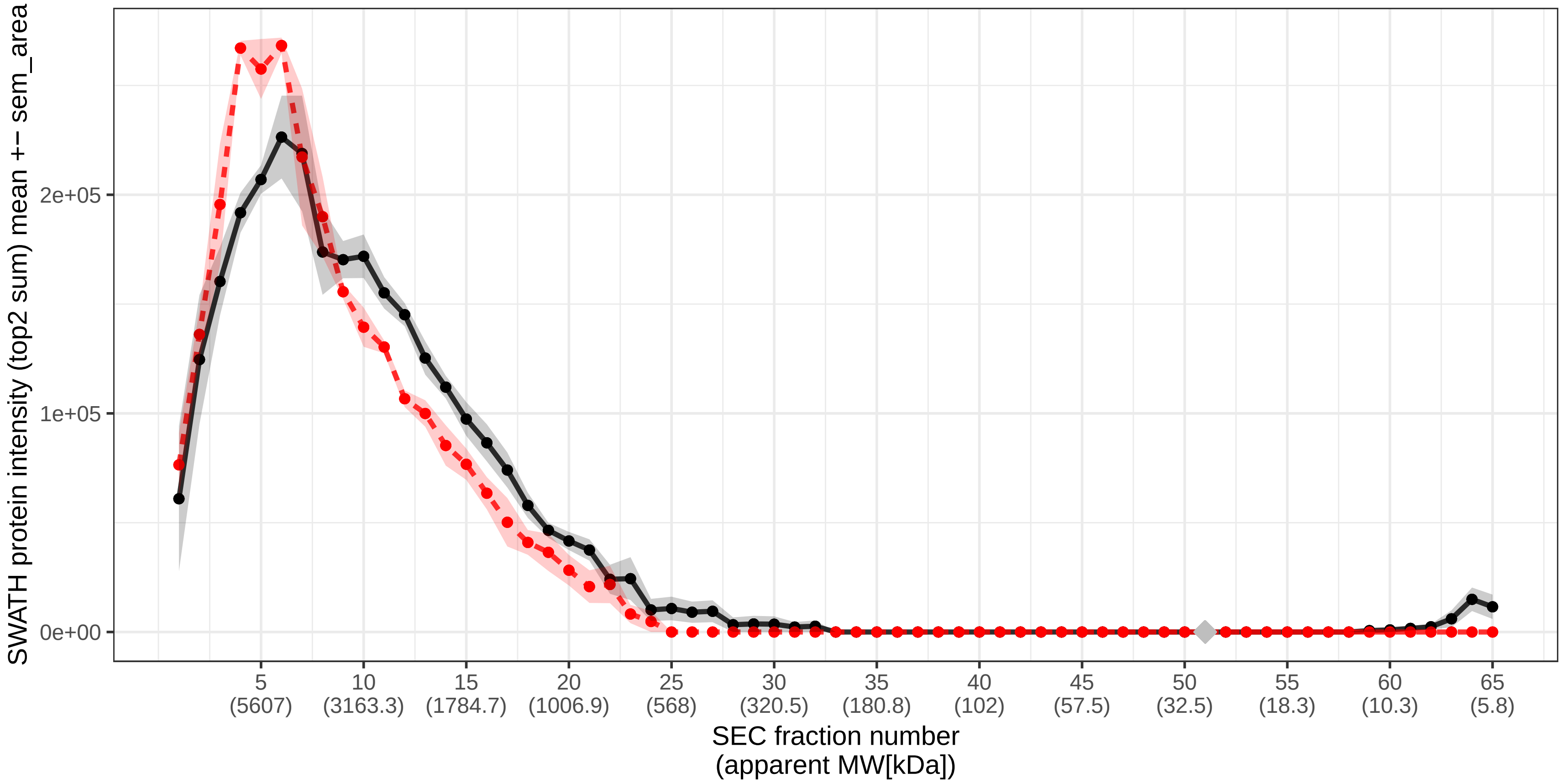

Supplement: Data S1. SEC-SWATH-MS Protein Chromatograms, Related to Figure 1 [file mmc6.zip › SECchrom_Q969M3_YIPF5_HUMAN_YIPF5_FINGER5_YIP1A_PP12723_SB1.pdf]
